# Electrical exposure analysis of galvanic-coupled intra-body communication based on the empirical arm models

**DOI:** 10.1186/s12938-018-0473-9

**Published:** 2018-06-05

**Authors:** Yue-Ming Gao, Heng-fei Zhang, Shi Lin, Rui-Xin Jiang, Zhi-Ying Chen, Željka Lučev Vasić, Mang-I Vai, Min Du, Mario Cifrek, Sio-Hang Pun

**Affiliations:** 1 College of Physics and Information Engineering, Fuzhou University, Fuzhou, 350116 China; 2 Key Lab of Medical Instrumentation & Pharmaceutical Technology of Fujian Province, Fuzhou, 350116 China; 3 Faculty of Electrical Engineering and Computing, University of Zagreb, Zagreb, Croatia; 4 State Key Laboratory of Analog and Mixed Signal VLSI, University of Macau, Macau, 999078 China; 5 Department of Electrical and Computer Engineering, Faculty of Science and Technology, University of Macau, Macau, 999078 China; 6 School of Electrical Engineering & Automation, Xiamen University of Technology, Xiamen, Fuzhou, 361024 China; 7 Key Lab of Eco-Industrial Green Technology of Fujian Province, Nanping, China

**Keywords:** Galvanic-coupled intra-body communication, Empirical arm models, ICNIRP guidelines, Electric field intensity, SAR, Exposure restrictions

## Abstract

**Background:**

Intra-body communication (IBC) is one of the highlights in studies of body area networks. The existing IBC studies mainly focus on human channel characteristics of the physical layer, transceiver design for the application, and the protocol design for the networks. However, there are few safety analysis studies of the IBC electrical signals, especially for the galvanic-coupled type. Besides, the human channel model used in most of the studies is just a multi-layer homocentric cylinder model, which cannot accurately approximate the real human tissue layer.

**Methods:**

In this paper, the empirical arm models were established based on the geometrical information of six subjects. The thickness of each tissue layer and the anisotropy of muscle were also taken into account. Considering the International Commission on Non-Ionizing Radiation Protection (ICNIRP) guidelines, the restrictions taken as the evaluation criteria were the electric field intensity lower than 1.35 × 10^4^ *f* V/m and the specific absorption rate (SAR) lower than 4 W/kg. The physiological electrode LT-1 was adopted in experiments whose size was 4 × 4 cm and the distance between each center of adjoining electrodes was 6 cm. The electric field intensity and localized SAR were all computed by the finite element method (FEM). The electric field intensity was set as average value of all tissues, while SAR was averaged over 10 g contiguous tissue. The computed data were compared with the 2010 ICNIRP guidelines restrictions in order to address the exposure restrictions of galvanic-coupled IBC electrical signals injected into the body with different amplitudes and frequencies.

**Results:**

The input alternating signal was 1 mA current or 1 V voltage with the frequency range from 10 kHz to 1 MHz. When the subject was stimulated by a 1 mA alternating current, the average electric field intensity of all subjects exceeded restrictions when the frequency was lower than 20 kHz. The maximum difference among six subjects was 1.06 V/m at 10 kHz, and the minimum difference was 0.025 V/m at 400 kHz. While the excitation signal was a 1 V alternating voltage, the electric field intensity fell within the exposure restrictions gradually as the frequency increased beyond 50 kHz. The maximum difference among the six subjects was 2.55 V/m at 20 kHz, and the minimum difference was 0.54 V/m at 1 MHz. In addition, differences between the maximum and the minimum values at each frequency also decreased gradually with the frequency increased in both situations of alternating current and voltage. When SAR was introduced as the criteria, none of the subjects exceeded the restrictions with current injected. However, subjects 2, 4, and 6 did not satisfy the restrictions with voltage applied when the signal amplitude was ≥ 3, 6, and 10 V, respectively. The SAR differences for subjects with different frequencies were 0.062–1.3 W/kg of current input, and 0.648–6.096 W/kg of voltage input.

**Conclusion:**

Based on the empirical arm models established in this paper, we came to conclusion that the frequency of 100–300 kHz which belong to LF (30–300 kHz) according to the ICNIRP guidelines can be considered as the frequency restrictions of the galvanic-coupled IBC signal. This provided more choices for both intensities of current and voltage signals as well. On the other hand, it also makes great convenience for the design of transceiver hardware and artificial intelligence application. With the frequency restrictions settled, the intensity restrictions that the current signal of 1–10 mA and the voltage signal of 1–2 V were accessible. Particularly, in practical application we recommended the use of the current signals for its broad application and lower impact on the human tissue. In addition, it is noteworthy that the coupling structure design of the electrode interface should attract attention.

## Background

Intra-body communication (IBC) uses the human body as a medium for electrical signals transmission [1, 2]. It has the advantages of high stability, low power consumption, easy connectivity, better anti-noise performance, and less radiation [3] and has become one of the physical layer standards recommended by IEEE 802.15.6 [4]. Owing to the fact that the human body is a complex organism composed of multiple tissue layers, it is hard for IBC studies to establish an effective equivalent model of local portions of the body or the whole body. The existing approaches of human body channel modeling mainly include the electromagnetic field numerical method [5, 6], the equivalent circuit model method [7, 8] and the phantom model method [9, 10]. Based on the appropriately constructed models, the human body channel transmission characteristics aim at optimizing the communication frequency and signal amplitude by comparing simulation with the in vivo experiment results, which provide a theoretical basis for the design of an IBC transceiver. However, the existing general models constructed by those methods are simple cylinder equivalents of the human body, which lack the specific parameters of models. In the meanwhile, since the model abstracted from a specific sample or a specific experimental human body can only characterize individual characteristics, it has great particularity and contingency, and cannot be used as an experimental benchmark model to study the universality of the human body channel. Therefore, it is necessary to find the proper balance between the simple cylinder model and the complex special individual model. Besides, there are few electrical safety regulations of the IBC signals applied to the human body. When the signals interact with the organism, part of the energy will be absorbed by the tissues, which may generate additional heat or change the electromagnetic field within the organism [11]. Research organizations around the world have mainly focused on the 50 Hz or higher frequencies of wireless communication and there are few investigations in the IBC frequency band. Therefore, it is significant to address this important exposure restrictions on the electrical IBC signals acting on the human body in terms of field intensity and frequency.

Although there are few studies on the exposure restrictions of the electrical IBC signals, study of the signal distribution and potential amplitude are involved in the study of human channel modeling. A five-layer concentric cylinder equivalent to the human arm was designed by Callejón et al. [5], and when a 1 mA alternating current was injected to the body, the current density distribution of each tissue layer was studied. Swaminathan et al. [12] analyzed the signal distribution at specific parts surrounding the excitation area along with the period of exposure. Lučev et al. [13] simplified the human arm to a four-layer concentric cylinder with a radius of 5 cm and a length of 45 cm, and they studied the proportion of current density distribution in different tissues. The results showed that the current density in the muscle layer is the largest. Based on the special working conditions of galvanic-coupled IBC, our research team [14] simplified the human arm model to a four-layer concentric cylinder and studied the change of the current density distribution in each tissue layer when the muscle’s electrical conductivity was changed under different frequencies. Although some studies have been conducted on the experiments of the IBC electrical signal on the human body, the exposure restrictions of galvanic-coupled IBC signals acting on the body was not considering under international guidelines systematically.

In this paper, based on the physiological information of each experimental subject, the upper and lower arms of the subjects were deemed to be equivalent to two circular truncated cones, which were used to construct the empirical arm models of all subjects. The thickness of each tissue layer and the anisotropy of muscle were also taken into account. The electric field intensity of < 1.35 × 10^4^ *f* V/m and SAR of < 4 W/kg in the 2010 ICNIRP guidelines, have been taken as the evaluation criteria. The electric field intensity can be obtained by using the finite element method (FEM), and the SAR can be calculated indirectly by the FEM, where the electric field intensity was the average value of all tissues, the localized SAR was averaged in 10 g continuous tissue. Then the calculated electric field intensity and SAR were compared with the ICNIRP guidelines to address the electrical exposure restrictions of galvanic-coupled IBC signals with different intensities and different frequency.

## Methods

### Geometry modeling

In studies on IBC modeling, an electrical signal is applied to the human body through the electrodes that are in direct contact with the skin. At the contact surface of the electrodes and skin, an ionic current is transformed to an electronic current or vice versa [15]. Hence the skin plays a vital role in the signal transmission. In addition, fat, muscle, and bone also have a huge impact on the transmission of electrical signals [16]. Therefore, we constructed a geometric model of the human arm, including skin, fat, muscle, and bone layers.

Six subjects (two female and four male) with different physiological characteristics were selected. The weight, fat percentage, and muscle percentage for each subject were measured by using PICOOC Latin Smart body scale (PICOOC Inc., Beijing, China). In addition, the geometric information of each subject arm was measured. According to the body mass index (BMI) standard [17], the subjects were divided into three types: under weight (BMI < 18.5), normal weight (BMI 18.5–25), and over weight (BMI ≥ 25). The physiological information of all subjects are listed in Table 1.

**Table 1:** Physiological information of the six subjects

Subject	Gender	Age	Height (cm)	Weight (kg)	BMI value	Fat percentage (%)	Muscle percentage (%)	Bone mass (kg)	Brachium (cm)	Arm circumference at 5 cm (cm)	Arm circumference at 15 cm (cm)	Arm circumference at 35 cm (cm)	Type
1	Female	26	163	44.10	16.6	20.9	74.6	2.0	48	14.0	20.5	21.5	Under weight
2	Male	26	168	49.96	17.7	13.3	82.1	2.2	45	15.5	21.0	22.0	Under weight
3	Female	26	160	54.78	21.4	29.0	66.9	2.2	45	16.0	21.0	25.0	Normal weight
4	Male	25	165	57.72	21.2	17.2	78.6	2.5	46	16.0	24.5	26.0	Normal weight
5	Male	26	178	67.80	21.4	15.1	80.5	2.8	48	17.0	24.5	26.5	Normal weight
6	Male	40	175	85.44	27.9	25.6	70.7	3.2	46	18.0	24.5	28.0	Over weight

**Fig. 1:**
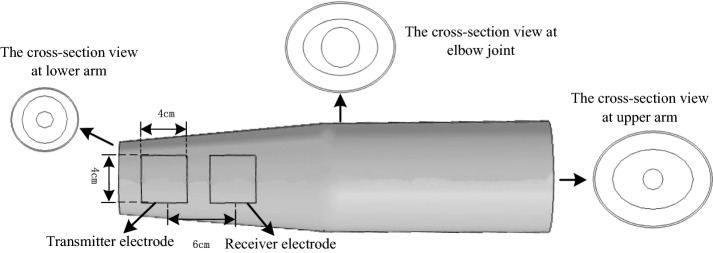
The empirical equivalent arm model and the electrode configurations

Based on our previous work, the human arm was divided into four layers: skin, fat, muscle, and bone [18], here the upper and lower arms were deemed to be equivalent to two elliptical cylinders, respectively. Because the thickness of the skin layer of different individuals is similar, the thickness of skin for all subjects was set to 1.5 mm in this paper [18]. According to the arm circumference, fat percentage and muscle percentage of subjects listed in Table 1, the tissue equivalent geometric parameters of each subject can be obtained, as shown in Table 2 where a and b represent the long semi axis and the short semi axis of the elliptical cylinder, respectively. Parameters d(a) and d(b) represent the thickness of tissues in the long semi axis and the short semi axis of the elliptical cylinder, respectively. Based on these parameters of Table 2 the personalized equivalent arm models of all subjects can be constructed. We adopted the physiological electrode LT-1 in our experiments whose size was 4 × 4 cm and the distance between each center of adjoining electrodes was 6 cm. Both the empirical equivalent arm model and the electrode configurations are shown in Fig. 1.

**Table 2:** Equivalent geometric tissue parameters for each subject

Subject		Upper arm (cm)				Elbow joint (cm)				Lower arm (cm)			
		a	d(a)	b	d(b)	a	d(a)	b	d(b)	a	d(a)	b	d(b)
1	Bone	0.573	0.573	0.506	0.506	1.162	1.162	0.894	0.894	0.513	0.513	0.481	0.481
	Muscle	3.045	2.472	1.859	1.353	2.848	1.686	1.661	0.767	1.978	1.465	1.424	0.943
	Fat	3.850	0.805	2.350	0.491	3.600	0.752	2.100	0.439	2.350	0.372	1.650	0.226
2	Bone	0.578	0.578	0.522	0.522	1.163	1.163	0.908	0.908	0.520	0.520	0.485	0.485
	Muscle	3.338	2.76	2.298	1.776	3.078	1.915	2.037	1.129	2.254	1.734	1.604	1.119
	Fat	3.850	0.512	2.650	0.36	3.550	0.472	2.350	0.313	2.600	0.346	1.850	0.246
3	Bone	0.580	0.580	0.521	0.521	1.140	1.140	0.886	0.886	0.529	0.529	0.484	0.529
	Muscle	3.090	2.51	2.094	1.573	2.380	1.24	1.491	0.605	1.882	1.353	1.384	0.9
	Fat	4.350	1.26	2.950	0.856	3.350	0.97	2.100	0.609	2.650	0.768	1.950	0.566
4	Bone	0.627	0.627	0.611	0.611	1.383	1.383	0.924	0.924	0.611	0.611	0.582	0.582
	Muscle	3.685	3.058	2.774	2.163	3.602	2.219	2.443	1.519	2.194	1.583	1.615	1.033
	Fat	4.450	0.765	3.350	0.576	4.350	0.748	2.950	0.507	2.650	0.456	1.950	0.335
5	Bone	0.656	0.656	0.656	0.656	1.191	1.191	0.930	0.930	0.622	0.622	0.592	0.592
	Muscle	3.778	3.122	2.954	2.298	3.693	2.502	2.504	1.574	2.356	1.734	1.783	1.191
	Fat	4.450	0.672	3.475	0.521	4.350	0.657	2.950	0.446	2.775	0.419	2.100	0.317
6	Bone	0.674	0.674	0.639	0.639	1.161	1.161	1.009	1.009	0.610	0.610	0.583	0.583
	Muscle	3.497	2.823	2.790	2.151	3.236	2.075	2.195	1.186	2.195	1.585	1.674	1.091
	Fat	4.700	1.203	3.750	0.96	4.350	1.114	2.950	0.755	2.950	0.755	2.250	0.576

### Simulation platform of galvanic coupled IBC

In galvanic-coupled IBC, a weak current or voltage signal is differentially sent using two transmitter and two receiver electrodes [19]. The simulation platform was developed and analyzed using the AC/DC Module, Electric Currents Physics, in COMSOL 5.2 Multiphysics Software (COMSOL Inc., Stockholm, Sweden). The control equations for the galvanic-coupled IBC under a quasi-static approximation field are

1 $$\left\{ {\begin{array}{*{20}l} {\nabla \cdot J = Q_{j} } \\ {J = (\sigma + j\omega \varepsilon_{0} \varepsilon_{r} )E + J_{e} } \\ {E = - \nabla V} \\ \end{array} } \right.$$

where J is the current density of body, *Q*_j_ is the quantity of electric charge, *J*_e_ is the density of current source, *σ* is the electrical conductivity, ω is the angular frequency, *ɛ*_0_ is the permittivity of vacuum, *ɛ*_r_ is the relative permittivity of the medium, *E* is the electric field intensity, V is the electric potential.

According to Dirichlet boundary conditions, the input electrical signal at the surface transmitter electrodes is [20]

2 $$V = V_{0}$$

where *V*_*0*_ is the amplitude of voltage applied to the human arm model by the transmitter electrodes.

The adjacent layers of the tissues, as well as the contact surface between the electrode and skin, have boundary conditions that satisfy the conditions of current continuity and voltage continuity:

3 $$\left\{ {\begin{array}{*{20}c} {V_{i} = V_{i - 1} } \\ {J_{i} = J_{i - 1} } \\ \end{array} } \right.$$

where *V*_*i−*1_ and *V*_*i*_ are the voltage between two adjacent layers, *J*_*i*−1_ and *J*_*i*_ represent the current density between two adjacent layers, and *i* is the number of layers, for values of 2, 3, and 4, respectively.

Then, in the material setting, because the IBC technology uses the human body as a conductive medium [1, 2], the model should contain the dielectric properties of human tissue. In addition, as mentioned in the literature [13], muscle fibers are stimulated by an external excitation source, the propagation velocity of electrical signal in different directions is different, that is, muscle has anisotropic properties. Thus, the conductivity of the muscle layer need to be anisotropic in the simulation environment. The dielectric properties of biological tissues at several frequencies are from Gabriel [21], as shown in Table 3.

**Table 3:** Dielectric parameters of different tissues

*f* (kHz)	Dry skin		Wet skin		Fat		Muscle			Bone	
	*σ* (S/m)	*ɛ*_r_	*σ* (S/m)	*ɛ*_r_	*σ* (S/m)	*ɛ*_r_	Transverse *σ* (S/m)	Longitudinal *σ* (S/m)	*ɛ*_r_	*σ* (S/m)	*ɛ*_r_
10	0.00020	1133.6	0.0029	29010	0.0238	1085.3	0.3408	1.022	25909	0.0204	521.64
100	0.00045	1119.2	0.0658	15357	0.0244	92.885	0.3619	1.086	8089.2	0.0208	227.64
200	0.00105	1104.8	0.1135	8849.2	0.0246	56.015	0.3841	1.152	6377.7	0.0211	203.74
400	0.00305	1076.3	0.1635	4514.6	0.0248	38.134	0.4278	1.283	4339.3	0.0218	182.27
1000	0.0132	990.76	0.2214	1832.8	0.0251	27.222	0.5027	1.508	1836.4	0.0243	144.51

Finally, the study was implemented in the frequency domain, the frequency range was set from 10 kHz to 1 MHz. The mesh was divided into a tetrahedral mesh controlled by a physical field, a steady-state solver in the frequency domain was selected, and the MUMPS algorithm was used to solve the problem. Results can be exported to a data table or to two or three-dimensional images.

### ICNIRP guidelines

In this paper, the ICNIRP guidelines were applied to address the electromagnetic exposure restrictions of the galvanic-coupled IBC electrical signals. The ICNIRP guidelines divide the basic restrictions into occupational exposure and general public exposure according to different group characteristics [22]. The occupational exposure is set for occupational groups working in a controllable radiation zone, who are professionally trained to take appropriate measures to protect themselves. The general public exposure refers to the general population of different genders, ages and health statuses who do not undergo professional training to avoid radiation.

When a subject is exposed to a time-varying electromagnetic field, three different physical quantities work as the basic restrictions at different frequencies. When the frequency ranges from 1 Hz to 10 MHz, the main limiting physical quantity is the electric field intensity (*E*), and at 100 kHz to 10 GHz, the main limiting physical quantity is the SAR, the last quantity is the power density (*S*), used for measured frequencies that are the highest: 10–300 GHz [22].

In addition, the 2010 ICNIRP guidelines (ICNIRP 2010) updates the low-frequency part of the 1998 guidelines (ICNIRP 1998). In particular, the ICNIRP 2010 replaces the current density with the electric field intensity as a new restriction. In the meanwhile, the SAR restrictions have remained the same as in ICNIRP 1998 guidelines. Therefore, Table 4 lists the basic restrictions for the electric field intensity at different frequencies under occupational exposure and general public exposure. And Table 5 lists the basic restrictions for the electric field intensity, while the part of current density is removed in the ICNIRP 2010.

**Table 4:** Basic restrictions for human exposure to time-varying electric fields [23]

Exposure characteristics	Frequency range	Internal electric field (V/m)
Occupational exposure		
The central nervous system tissue of the head	1–10 Hz	0.5/*f*
	10–25 Hz	0.05
	25–400 Hz	2 × 10^−3^ *f*
	400 Hz–3 kHz	0.8
	3 kHz–10 MHz	2.7 × 10^−4^ *f*
All tissues of head and body	1 Hz–3 kHz	0.8
	3 kHz–10 MHz	2.7 × 10^−4^ *f*
General public exposure		
The central nervous system tissue of the head	< 1 Hz	0.1/*f*
	1–4 Hz	0.01
	4 Hz–1 kHz	4 × 10^−5^ *f*
	1–100 kHz	0.4
	100 kHz–10 MHz	1.35 × 10^−4^ *f*
All tissues of head and body	1 Hz–3 kHz	0.4
	3 kHz–10 MHz	1.35 × 10^−4^ *f*

**Table 5:** Basic ICNIRP guideline restrictions for time-varying electromagnetic fields [22]

Exposure characteristics	Frequency range	Whole-body average SAR (W/kg)	Localized SAR (head and trunk) (W/kg)	Localized SAR (limbs) (W/kg)
Occupational exposure	100 kHz to 10 GHz	0.4	10	20
General public exposure	100 kHz to 10 GHz	0.08	2	4

Since the IBC technical application is aimed at the general group, we considered the general public exposure restrictions as a standard in this work. The signal frequency range of galvanic-coupled IBC was from 10 kHz to 1 MHz, hence the electric field intensity of < 1.35 × 10^−4^ *f* V/m at 10 kHz–1 MHz and SAR of < 4 W/kg at the frequency range of 100 kHz–1 MHz, were adopted as the evaluation criteria to explore the exposure restrictions of galvanic-coupled IBC signals acting on the human arm.

## Results

### The electric field intensity

Based on a simulation platform of galvanic-coupled IBC, the electric field intensity of all model subjects can be obtained. By comparing with the restrictions of the electric field intensity in ICNIRP guidelines, we investigated and concluded the moderate exposure restrictions of the electrical IBC signals according to the experiments of different frequencies and different signal intensities (including current and voltage) on the electric field intensity.

**Fig. 2:**
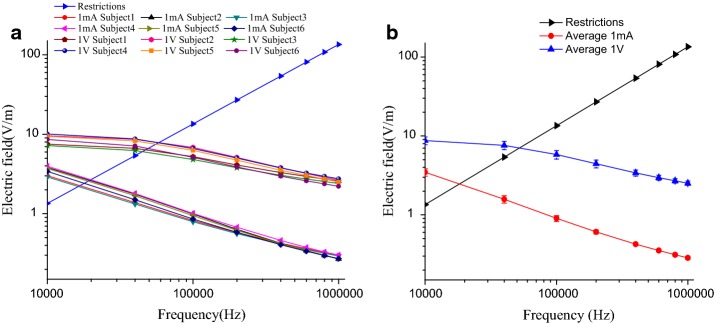
Comparison of electric field and basic restrictions. **a** Electric field of different subjects at different frequencies, **b** the average of subjects of electric field at different frequencies

To discuss the influence of electric field intensity on the human arm under different frequencies, the signal frequency was set to 10 kHz–1 MHz, a 1 V alternating voltage and a 1 mA alternating current were then applied to the six human arm models, respectively. And then the values of the electric field intensity E (unit: V/m) under different frequency were obtained by FEM. As shown in Table 4, when the body is exposed to time-varying electric fields and magnetic fields, the basic restrictions of the electric field intensity at the frequency of 10 kHz to 1 MHz are lower than 1.35 × 10^−4^ *f* V/m. Therefore, in the frequency range of galvanic-coupled IBC, the restrictions of the electric field intensity in the human body are 1.35–135 V/m. A comparison of the restrictions and the electric field intensity of subjects under different frequencies is shown in Fig. 2a, and the average electric field intensity of subjects under different frequencies is shown in Fig. 2b.

As shown in Fig. 2a, the electric field intensity of each subject is reduced with the increase of signal frequency. Based on the FEM, the average electric field intensity *E* of the six subjects at different frequencies was calculated. A comparison of the restrictions and the average electric field intensity of subjects under different frequencies is shown in Fig. 2b. It can be seen that when the subject was stimulated by a 1 mA alternating current, the curve of the electric field intensity trend to decrease gradually with the increase of frequency, whose intersectional point with the restrictions curve was at 20 kHz nearby. That means, the average electric field intensity of all subjects exceeded restrictions when the frequency was lower than 20 kHz. The maximum difference between six subjects was 1.06 V/m at 10 kHz, and the minimum difference was 0.025 V/m at 400 kHz. While the excitation signal was a 1 V alternating voltage, the curve of the electric field intensity trend to decrease gradually with the increase of frequency, whose intersectional point with the restrictions curve was at 50 kHz nearby. As the frequency increased beyond 50 kHz, the electric field intensity fell within the safety restrictions gradually. The maximum difference between the six subjects was 2.55 V/m at 20 kHz, and the minimum difference was 0.54 V/m at 1 MHz. In addition, differences between the maximum and the minimum at each frequency also decreased gradually with the frequency increased in both situations of alternating current and voltage.

**Fig. 3:**
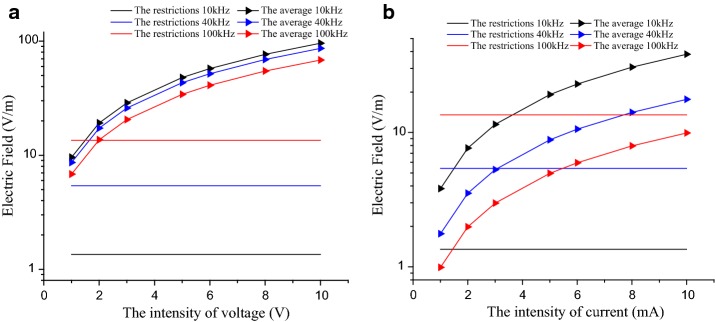
Relationship between the electric field and the exposure restrictions when the signal intensity is different. **a** Electric field at different signal intensities with the current excitation, **b** electric field at different signal intensities with the voltage excitation

Furthermore, it can be known that when the subject was stimulated by a 1 mA alternating current, the electric field intensity at the lower frequencies exceeded the restrictions while it didn’t at the higher frequencies. So, 10, 40, and 100 kHz which were assigned to the lower frequencies were chosen to explore the effects of electrical signals with different intensities on the electric field intensity. Two excitation sources were used: the AC voltage source, whose peak-to-peak value was 1–10 V, and the AC current source, whose intensity was 1–10 mA. The results are shown in Fig. 3.

With the increase of signal intensities, the electric field intensity also increased. At lower frequencies, such as 10 and 40 kHz, the electric field intensities were higher than the basic restrictions with the voltage signal of 1–10 V input. While at 100 kHz, the electric field intensities satisfied the restrictions only when the signal intensity is range from 1 to 2 V. However, with the excitation current of 1–10 mA, the electric field intensities at 10 kHz exceeded the restrictions. Only part of them exceeded the restrictions at 40 kHz and all of them satisfied the restrictions at 100 kHz. It can be known that the lower the frequency is, the greater the probability that the electric field intensity exceeds the basic restrictions. In addition, at the same frequency, the electric field intensity increased with the increase of the current intensity. That means, the higher the alternating current intensity is, the greater the probability that the electric field intensity exceeds the basic restrictions at the same frequency. Therefore, to avoid the harm to human body in galvanic coupled IBC, we should use electrical signals with low intensity and high frequency.

### SAR

In this section, the SAR is used as the exposure restrictions of galvanic-coupled IBC and the relationships between the SAR and different signal frequencies and different signal intensities are explored.

SAR indicates the degree of absorption or consumption of electromagnetic energy per unit mass in biological tissue, which is mainly used to measure the degree of interaction between electromagnetic waves and tissue [24]. It can be described by the following equation [25]:

4 $$SAR(i,j,k) = \frac{1}{2\rho (i,j,k)}\left[ \begin{aligned} \sigma_{x} (i,j,k) \cdot \left| {E_{x} (i,j,k)} \right|^{2} + \sigma_{y} (i,j,k) \cdot \left| {E_{y} (i,j,k)} \right|^{2} \hfill \\ + \sigma_{z} (i,j,k) \cdot \left| {E_{z} (i,j,k)} \right|^{2} \hfill \\ \end{aligned} \right]$$

where *E*_*x*_, *E*_*y*_, and *E*_*z*_ are the electric field component along the *x*, *y*, and *z* axes, respectively; *σ*_*x*_(*i*, *j*, *k*), *σ*_*y*_(*i*, *j*, *k*) and *σ*_*z*_(*i*, *j*, *k*) are the conductivity values of each point along the *x*, *y*, and *z* directions, respectively; and *ρ*(*i*, *j*, *k*) is the density of tissue.

The SAR cannot be directly calculated in COMSOL, so Eq. (4) was used to calculate the localized SAR of arm. The ICNIRP guidelines specify that the localized SAR of the limbs is not lager than 4 W/kg per 10 g continuous tissue at the frequency of 100 kHz–10 GHz. Thus, we obtained the maximum SAR through Eq. (4) and then calculate the summation of maximum SAR in 10 g continuous tissue. By interfacing COMSOL with MATLAB (Math Works, USA, http://www.mathwork.com), the calculation process can be completed by using MATLAB, the steps were as follows:

1. The point coordinates set of each tissue layer were obtained. Since there are critical surfaces between tissue layers, we stipulated that the point on critical surface is assigned into the inner tissue, which is used for obtaining the area of tissues, *S*_*bone*_, *S*_*muscle*_, *S*_*fat*_, *S*_*skin*_.
2. The FEM was used to calculate the electric field intensity component along the directions of *x*, *y* and *z*, that is *E*_*x*_, *E*_*y*_, and *E*_*z*_, the *σ*_*x*_(*i*, *j*, *k*), *σ*_*y*_(*i*, *j*, *k*) and *σ*_*z*_(*i*, *j*, *k*) can be derived from Gabriel [20], the *ρ*(*i*, *j*, *k*) is equivalent density of whole body in this work [26], whose value is 1000 kg/m^3^.
3. These parameters were substituted into Eq. (4), and the maximum SAR is obtained and then localized SAR can be calculated as the summation of maximum SAR in 10 g continuous tissue.

In galvanic coupling IBC, the electrical signals are mainly transmitted through the skin, fat and muscle layers, whose density is 1109, 911 and 1090 kg/m^3^ respectively, referring to the website [27]. However, for different types of the bone the density is different, such as bone cancellous 1178 kg/m^3^, bone cortical 1908 kg/m^3^, bone marrow (red) 1029 kg/m^3^ and bone marrow (yellow) 980 kg/m^3^. Most of these tissues’ densities are all close to 1000 kg/m^3^ except the bone cortical. But the volume of the bone cortical and current flow through it are low, so we set 1000 kg/m^3^ as the average density of the bone layer in the model.

**Fig. 4:**
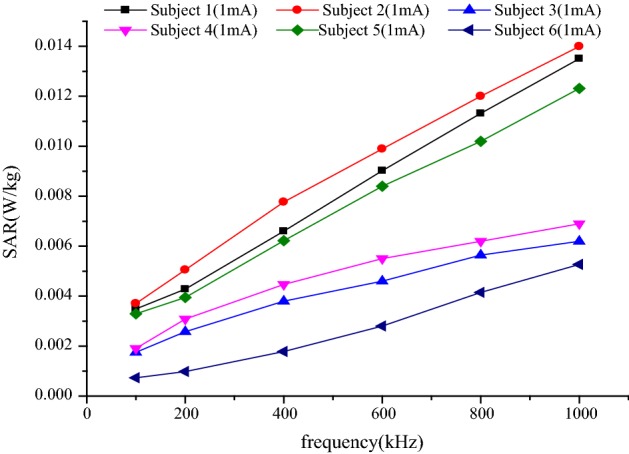
Localized SAR of the human arm under 1 mA excitation at different signal frequencies

The localized SAR of each subject was calculated at the frequency of 100 kHz–1 MHz. Results were shown in Fig. 4. The maximum localized SAR of different subjects was 0.014 W/kg with the 1 mA alternating current, whose value was much lower than the restrictions. In addition, as frequency increased, the SAR showed an approximately linearly increasing trend. So, when the frequency was higher, the SAR may be higher than the basic restrictions referring to Table 5.

To analyze the effects of different signal intensities on the human arm, the higher frequencies of 100 kHz, 400 kHz, and 1 MHz were selected. The intensities of the input alternating current ranged from 1 to 10 mA, and intensities of the input alternating voltage were 1–10 V, respectively. It can be seen from Fig. 4 that the SAR of subject 2 was the highest at the same signal intensity and that of subject 6 was the lowest, the SARs of subject 1, subject 3, subject 4, and subject 5 were between the maximum and the minimum. Hence SAR values of subjects 2 and 6, as well as of subject 4, were further analyzed in more details.

From Table 6, it can be seen that as the signal intensity increased, the localized SAR of all subjects increased. The SAR of all subjects were not higher than 4 W/kg with signal intensity of 1–10 mA. The maximum difference of subjects was 1.300 W/kg when the signal frequency was 1 MHz and the intensity was 10 mA; the minimum difference was 0.062 W/kg with the signal frequency of 400 kHz and the signal intensity of 3 mA. However, in the case of voltage signal, when the intensity was ≥ 3 V and the frequency was > 400 kHz, the local SAR value of subject 2 did not qualify the basic restrictions, that of subject 4 exceeded the basic restrictions with the intensity ≥ 6 V and the frequency > 400 kHz, while subject 6 did not satisfy the range of restrictions with the intensity ≥ 10 V and the frequency > 400 kHz. The maximum difference of subjects was 6.096 W/kg when the signal frequency was 1 MHz and the intensity was 10 V, and the minimum difference was 0.648 W/kg with the signal frequency of 400 kHz and the signal intensity of 1 V.

**Table 6:** SAR of the human arm at different signal intensities (W/kg)

Signal intensity	Subject 2			Subject 4			Subject 6		
	100 kHz	400 kHz	1 MHz	100 kHz	400 kHz	1 MHz	100 kHz	400 kHz	1 MHz
3 mA	0.08	0.11	0.20	0.07	0.10	0.12	0.02	0.05	0.09
6 mA	0.33	0.43	0.81	0.27	0.38	0.62	0.07	0.18	0.34
10 mA	0.90	1.19	2.24	0.75	1.05	1.73	0.18	0.50	0.94
1 V	0.77	1.22	2.31	0.58	0.85	1.55	0.12	0.34	0.69
3 V	1.24	2.33	*4.02*	0.78	1.25	1.97	0.15	0.64	1.36
6 V	1.87	*4.56*	*6.72*	1.00	2.49	*4.03*	0.27	1.55	2.71
10 V	2.77	*6.32*	*10.86*	1.35	3.83	*6.32*	0.41	2.79	*4.76*

*Note* the values in italics emphasise that the signal intensities exceed the 4 W/kg restriction

## Discussion

In this study, we investigated the performances of the galvanic-coupled IBC signals with different frequencies and different intensities in order to address the applicable exposure restrictions.

When the electric field intensity was used as the evaluation criterion, the average electric field intensity of six subjects exceeded the range of exposure restrictions with 1 mA current or 1 V voltage applied at low frequencies. To be exact, the safety frequency restriction is 20 kHz for current signal and 50 kHz for voltage signal (Fig. 2). The differences in the average electric field intensity of subjects at different frequencies were 0.025–1.06 V/m of current input, the differences were 0.54–2.55 V/m of voltage input. Thus, the electric field intensity generated by the lower frequency signal applied to the body was easier to exceed the basic restrictions and different individuals have different extents of reaction with the excitation signals (with the same intensity and frequency) input. In order to discuss the influence of different signal intensity to the electric field intensity, the lower frequencies (10, 40, and 100 kHz) were chosen (Fig. 3). The electric field intensities of most points did not meet the range of exposure restrictions with the input voltage signal of 1–10 V. As for current signal of 1–10 mA, part of the electric field intensities was higher than the restrictions at 40 kHz while all points satisfied the restrictions at 100 kHz.

Also, the tendency can be concluded from Fig. 3 that the electric field intensities increased with signal intensities increased. In summary, for current signals, the intensities restrictions of 1–3 mA was accessible with the frequency restrictions higher than 50 kHz. The restrictions range of voltage signals was 1–2 V with the frequency restrictions higher than 100 kHz. With the frequency rising, both current and voltage signal intensity restrictions range would be wider.

When SAR was adopted as the evaluation criterion, none of the subjects exceeded that restrictions with current input. However, subjects 2, 4, and 6 did not qualify the range of restrictions with voltage applied when the signal intensity is then ≥ 3, 6, and 10 V, respectively (for frequency ≥ 400 kHz) (italic values in Table 6). It can be known that the body’s SAR was not easier to meet the range of restrictions with the signal of higher intensity and higher frequency input, which may have a negative impact on the human body. And the SAR differences for subjects under different frequencies were 0.062–1.3 W/kg of current input, and the differences were 0.648–6.096 W/kg of voltage input.

According to the localized SAR of the body under different signal frequencies and different signal intensities, it is necessary to use an excitation signal with lower intensity and lower frequency in the galvanic-coupled IBC. To be more exact, for current signals, less restrictions were required with the intensities of 1–10 mA, while the intensity restrictions range of voltage signals was 1–3 V with the frequency restrictions lower than 400 kHz. With the frequency falling, the current intensity restrictions range would be wider.

In summary, the constant-current source has more broadly applicability than the constant-voltage source due to its intensity restriction range is wider. We comprehensively considered the frequency of 100–300 kHz that belong to LF (30–300 kHz) according to the ICNIRP guidelines was reasonable for application in view of the electric field intensities and the SAR experiments, which also provides more choices for both intensities of current and voltage signals. On the other hand, it provides great convenience for the design of transceiver hardware and artificial intelligence application. Therefore, to avoid over exposure to the human body in vivo experiments and miscellaneous design, we considered the restrictions that the current signal with intensity of 1–10 mA and the voltage signal with intensity of 1–2 V were accessible, both of them have frequency restrictions of 100–300 kHz. In particular, we recommended the current signals in practical application.

## Conclusion

In this paper, the empirical arm models were constructed. The electric field intensity and SAR restrictions from the ICNIRP guidelines were adopted as evaluation criteria, and FEM was used to analyze the performances of weak electrical signals in galvanic-coupled IBC on human arm models. The empirical arm model simulation results showed that for current signals, the intensities restrictions of 1–3 mA were accessible with the frequency restrictions higher than 50 kHz. The restrictions range of voltage signals was 1–2 V with the frequency restrictions higher than 100 kHz. Besides, when the SAR was used as criterion, for current signals, less restrictions were required with the intensities of 1–10 mA, while the intensity restrictions range of voltage signals was 1–3 V with the frequency restrictions lower than 400 kHz. Therefore, based on the empirical arm model simulation results, we comprehensively considered the frequency of 100–300 kHz that belong to LF (30–300 kHz) according to the ICNIRP guidelines as the frequency restrictions to avoid harm to the human body, which provided more choices for both intensities of current and voltage signals as well. On the other hand, it also makes great convenience for the design of transceiver hardware and artificial intelligence application. With the frequency restrictions settled, the intensity restrictions that the current signal of 1–10 mA and the voltage signal of 1–2 V were accessible. Particularly, we recommended the current signals in practical application for its broad application. Our simulation study provides an applicability evaluation for IBC technology that can be used to design electronic devices and also promote application of IBC.
